# Erythematous Plaques and Tense Bullae in an Infant

**DOI:** 10.7759/cureus.16623

**Published:** 2021-07-25

**Authors:** Amanda Ederle, Cees T Whisonant, Hugh Nymeyer

**Affiliations:** 1 Internal Medicine, Baptist Health, Little Rock, USA; 2 Division of Plastic, Reconstructive, Hand and Burn Surgery, Department of Surgery, University of New Mexico Hospital, Albuquerque, USA; 3 Dermatology, University of Arkansas for Medical Sciences, Little Rock, USA

**Keywords:** bullous skin disease, blistering skin disease, pediatrics, pediatric dermatology, bullous pemphigoid

## Abstract

Although often thought of as a disease of the elderly, bullous pemphigoid is the second most common bullous disease in infants. Infantile bullous pemphigoid is extremely rare and may be easily confused with other skin diseases such as epidermolysis bullosa and chronic bullous disease of childhood. There appears to be a paucity of literature on unique clinical presentations of infantile bullous pemphigoid. In this report, we describe a case of infantile bullous pemphigoid, which presented with tense bullae in a widespread distribution, including many labial bullae. The rash initially began on this patient’s temples and ears four days prior. We believe this case will be of interest as it demonstrates a rare infantile disease with an unusual clinical presentation. It is important to consider infantile bullous pemphigoid in a patient presenting with tense bullae and initiate appropriate diagnostic studies.

## Introduction

Infantile bullous pemphigoid (BP) is a rare blistering skin disease. More commonly, BP is a disease of the elderly population. However, due to its rarity and lifelong remission with proper treatment in infants, it is vital to recognize these patients and initiate treatment early in the disease course. In this case report, we present the case of a five-month-old female infant with tense bullae scattered across her body. Proper treatment with oral prednisone, topical corticosteroids, and dapsone was started in this patient, and within one week, she had incredible improvement in her blisters. It is important for infantile BP to be high on the differential with blistering skin diseases in infants. 

## Case presentation

A five-month-old full-term female infant presented to the emergency department with a rapidly progressive blistering rash that started four days earlier. The rash first appeared around her temples and ears and spread over several days to involve her trunk and all four limbs.

**Figure 1:**
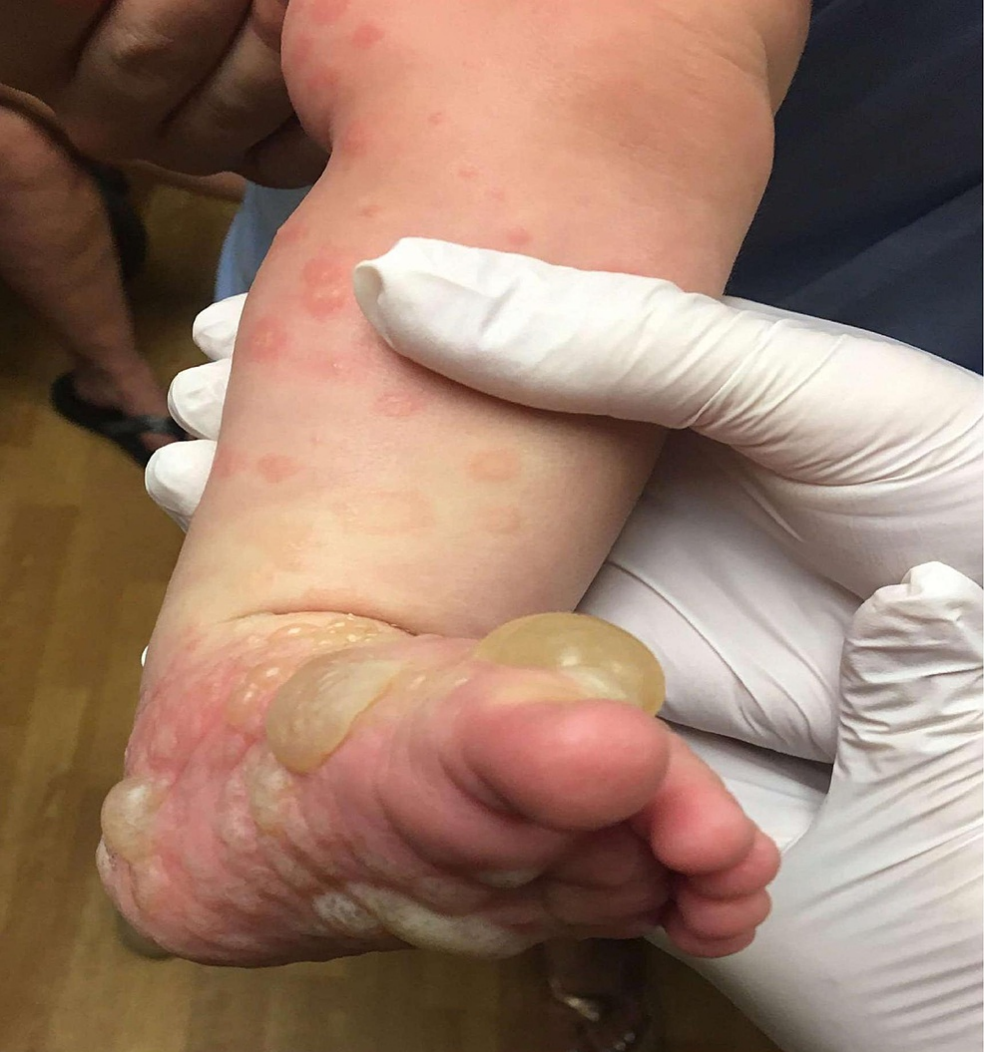
Clinical Image

Tense bullae appeared on her feet, genitals, and ears. On physical examination, erythematous round and annular plaques were present on the entire body. Tense vesicles and bullae were noted on the hands, feet, and labia (Figure 1). 

Her mother denied that the patient had a recent fever or medication use but did report that the patient recently had signs of an upper respiratory infection.

**Figure 2:**
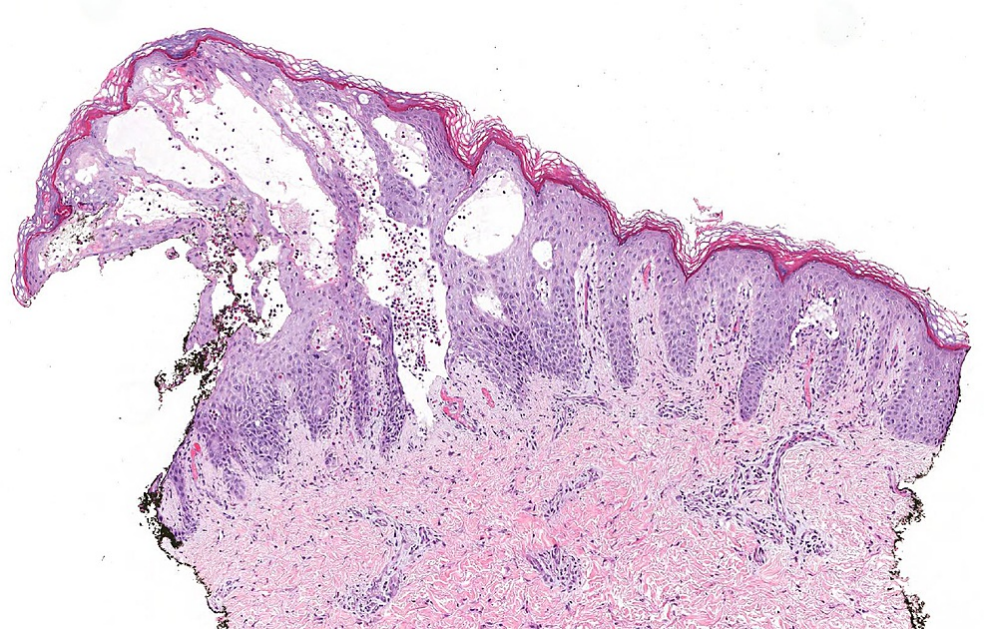
Hematoxylin and Eosin Stain (77X Magnification)

We obtained two punch biopsies from the patient's foot for hematoxylin and eosin (H&E) stain and direct immunofluorescence (DIF) after admission to the hospital. H&E showed a sub-epidermal blister with prominent eosinophilia consistent with the diagnosis of BP (Figure 2).

DIF showed deposition of IgG and C3 at the basement membrane zone. Plasma was sent for indirect immunofluorescence (IIF) and enzyme-linked immunosorbent assay (ELISA) testing. ELISA testing confirmed high levels of BP antigen-2 antibodies (204 U/mL) with normal levels of BP antigen-1 antibodies (4 U/mL). IIF performed on salt-split human skin showed deposition of IgG detectable at dilutions of 1:640 on the epidermal side of the split skin. There was no detectable IgA. These results were consistent with a diagnosis of BP. 

Our patient was admitted to the hospital and started on oral prednisone 2 mg/kg daily and topical halobetasol. At the next appointment, the patient was started on dapsone 0.5 mg/kg daily. One month later, physical examination revealed erythematous macules on the hands, feet, genitals, and buttocks but no vesicles or bullae. Dapsone was increased to 1 mg/kg daily. Subsequent follow-up appointments revealed continuation of the erythematous macules but without bullae. Daily prednisone was tapered two months after initial presentation and discontinued completely over the next two months. The patient has been continued on dapsone alone. We plan to continue dapsone for a total of six to nine months and then attempt to taper while monitoring for recurrent flares.

## Discussion

BP is an autoimmune blistering disease that classically appears as tense bullae and erythematous plaques widely disseminated on the head, trunk, and limbs [1,2]. Lesions may also be present on mucosal membranes including the oral cavity, genitals, and peri-anally with oral bullae occurring in 10-30% of patients [1]. BP is typically a disease of the elderly with the average age of diagnosis between 75 and 81 years [3]. It is typically a chronic disease with periods of exacerbation and remission often requiring lifelong immunosuppressive treatment. 

Despite its relative rarity, infantile BP has been reported, after chronic bullous disease of childhood, to be the second most common immunobullous disease in infants with more than 80 reported cases [4,5]. There is an unusual predominance of cases of BP before the first year of age, with a median age of diagnoses in infants of four months [6]. Although sharing many of the features of adult-onset BP, infantile BP shows more acral and facial involvement, characteristics seen in the patient here, and less genital involvement [6]. Genital involvement is reported in only 5% of infantile cases [6]. Infantile BP also has higher mean levels of circulating BP antigen-2 antibodies than adult-onset BP, a characteristic seen in the patient here [5]. Infantile BP occurs more suddenly and with greater severity than adult-onset BP. Despite this, reviews of the reported cases show that unlike adult-onset BP, infantile BP responds rapidly to treatment and nearly always goes into remission after a short course of immunosuppressive treatment [5].

Once one suspects BP clinically, it is important to obtain lesional and perilesional punch biopsies. Lesional biopsies are examined with H&E stains to look for sub-epidermal blistering with prominent eosinophilia. Perilesional biopsies are examined using DIF to look for linear deposition of IgG and C3 in the basement membrane zone. If desired, confirmatory testing can be obtained using a serum specimen for IIF and ELISA testing to identify circulating basement membrane antibodies.

The treatment of BP involves immunosuppressive therapy that usually starts with class I topical steroids or systemic corticosteroids. Transitioning to a steroid-sparing immunosuppressant agent such as mycophenolate mofetil, azathioprine, methotrexate, erythromycin, nicotinamide, intravenous immunoglobulin, or rituximab is important. Dapsone, in particular, has been used frequently in the pediatric BP population with particular effectiveness [5].

## Conclusions

This case highlights the importance of being clinically aware of BP in infants, which is more common than currently recognized. Diagnosis of infantile BP can be made in the same manner as in adults. Its presentation is similar to adults but with more involvement of the head and acral sites. Notably, it is also necessary to examine mucosal areas including the genitals and oral mucosa, as mucosal involvement is more common in infantile BP. It is important to be aware of the better prognosis seen in infantile BP, which can be expected to go into long-term remission after brief treatment with appropriate immunosuppressive agents.
